# Trends and Disparities in Hemorrhagic Stroke and Hypertension‐Related Mortality in the United States From 1999 to 2023: A CDC WONDER Database Analysis

**DOI:** 10.1002/brb3.70704

**Published:** 2025-07-21

**Authors:** Muhammed Ameen Noushad, Emer Mulholland, Jaisurya Jaisukhalal, Reem Abukhater, Wahhaj Qayyum, Munikaverappa Anjanappa Mukesh, Jovita Jerome D silva, Mubashir Tanvir Sheikh, Muhammad Muneeb Arshad, Naweed Ebrahim Essa, Eeshal Zulfiqar, Mushood Ahmed, Raheel Ahmed

**Affiliations:** ^1^ University Hospital Plymouth NHS Trust Plymouth UK; ^2^ RAK Medical and Health Sciences University Ras Al‐Khaimah UAE; ^3^ South Tyneside and Sunderland NHS Foundation Trust Sunderland UK; ^4^ Gastroenterology Cumberland Infirmary Carlisle UK; ^5^ University Hospital Birmingham, NHS Foundation Trust Birmingham UK; ^6^ Department of Medicine Dow University of Health Sciences Karachi Pakistan; ^7^ Department of Medicine Rawalpindi Medical University Rawalpindi Pakistan; ^8^ National Heart & Lung Institute Imperial College London London UK; ^9^ Department of Cardiology Royal Brompton Hospital London UK

**Keywords:** CDC WONDER, hypertension, mortality, stroke

## Abstract

**Background:**

Hemorrhagic stroke remains a major cause of mortality, with hypertension being a key modifiable risk factor. Despite advancements in management, demographic and geographic disparities persist. This study aims to analyze hemorrhagic stroke‐related mortality trends among hypertensive adults in the United States from 1999 to 2023, stratified by sex, race, and geographic location.

**Methods:**

We utilized death certificate data from the CDC WONDER database for individuals aged ≥ 25 years from 1999 to 2023. Crude mortality rates (CMR) and Age‐adjusted mortality rates (AAMRs) per 100,000 persons were calculated, and annual percentage change (APC) were determined using Joinpoint regression analysis.

**Results:**

From 1999 to 2023, a total of 372,922 deaths were identified related to hemorrhagic stroke and hypertension. The overall AAMR was 0.45 in 1999 and 6.88 in 2023, with no significant trend observed over the study period. Males consistently exhibited higher AAMRs than females (Males: 7.76 vs. Females: 6.06 in 2023). When stratified by race, the highest AAMR was observed in non‐Hispanic (NH) Black or African American populations, followed by NH other, Hispanic or Latino, and NH White populations (AAMR of 10.95, 8.20, 7.83, and 5.86, respectively, in 2023). Regionally, the highest mortality was observed in the West, followed by the South, the Midwest, and lastly, the Northeast (with values of 7.91, 7.76, 5.91, and 4.82, respectively, in 2023). Urban areas (6.79) exhibited consistently higher AAMRs than rural areas (6.13) from 1999 to 2020.

**Conclusion:**

Hemorrhagic stroke and hypertension‐related mortality remained stable in the United States from 1999 to 2023, with males, NH Black or African American populations, and the Western region exhibiting the highest AAMRs. These findings highlight the importance of improving hypertension management and addressing mortality disparities.

## Graphical abstract

Hemorrhagic stroke‐related mortality among hypertensive adults in the U.S. remained stable from 1999 to 2023, with no significant trends observed in age‐adjusted mortality rates (AAMRs).> Males consistently had higher mortality than females, and non‐Hispanic Black individuals experienced the highest AAMRs compared to other racial/ethnic groups. The highest mortality rates were observed in the Western region, followed by the South, Midwest, and Northeast. Urban areas had consistently higher AAMRs compared to rural areas over the study period.

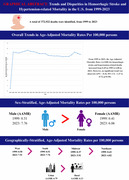

## 1 Introduction

Hemorrhagic stroke, characterized by bleeding within or around the brain, accounts for approximately 8%–15% of all stroke cases in the United States (Unnithan et al. 2025). There are two primary types of hemorrhagic strokes: intracerebral hemorrhage (ICH) and subarachnoid hemorrhage (SAH), with ICH being a more common subtype. ICH accounts for 10% of all strokes in the United States, with mortality as high as 50% within 1 month (Virani et al. 2020; Fogelholm et al. 2005; van Asch et al. 2010). Hypertension is a significant modifiable risk factor for hemorrhagic stroke, contributing to both its incidence and severity. Nearly half of US adults (47.7%) have hypertension, with prevalence increasing with age (Centers for Disease Control and Prevention 2024). It is estimated that 17%–28% of hemorrhagic strokes among hypertensive patients could be prevented with proper hypertension management (Woo et al. 2004; Wajngarten and Silva 2019).

Hypertension contributes to hemorrhagic stroke through several mechanisms. Prolonged hypertension weakens cerebral blood vessels, leading to the formation of microaneurysms and increasing their susceptibility to rupture (Woo et al. 2004; Sierra et al. 2011). In addition, elevated blood pressure exacerbates hematoma expansion, worsening mass effect and neurological deficits (Leasure et al. 2019). Hypertension‐induced endothelial dysfunction further disrupts the blood–brain barrier, increasing the likelihood of hemorrhage (Sierra et al. 2011). As a result, hypertensive patients who experience hemorrhagic stroke tend to have poorer clinical outcomes, including greater disability, higher mortality, and an elevated risk of recurrence (Woo et al. 2004; Dubow and Fink 2011).

While the relationship between hypertension and outcomes in hemorrhagic stroke is well established, certain populations across the United States remain disproportionately affected (Xian et al. 2014). Understanding these disparities is crucial for identifying high‐risk populations and assessing the impact of hypertension on hemorrhagic stroke mortality. In this study, we will analyze hemorrhagic stroke and hypertension related mortality trends in the United States using the CDC WONDER database from 1999 to 2023. Our analysis will be stratified by age, sex, race, and geographical location to identify populations at heightened risk.

## 2 Methods

### 2.1 Study Setting

For this study, we utilized mortality data from the National Center for Health Statistics (NCHS), accessed through the Centers for Disease Control and Prevention Wide‐Ranging Online Data for Epidemiologic Research (CDC WONDER) database (Centers for Disease Control and Prevention n.d.). We analyzed annual mortality trends using death certificate data from 1999 to 2023 for individuals aged 25 years and older in the United States. This dataset includes both underlying causes of death and contributing conditions, along with patient demographic information. We identified hypertension and hemorrhagic stroke‐related deaths using the Multiple Cause of Death Public Use File and the International Classification of Diseases, 10th Revision (ICD‐10) codes. Hypertension‐related deaths were classified under ICD‐10 codes I10–I15, while hemorrhagic stroke‐related deaths were categorized under I60–I62. These ICD‐10 codes are validated and have been used in prior studies (Doddi et al., 2024; Rethy et al. 2020). Individuals with both conditions listed as either the primary cause of death or a contributing factor were included in our analysis. The data used in this study are publicly available and de‐identified, ensuring no risk of confidentiality breaches. Therefore, Institutional Review Board (IRB) approval was not required. This study adhered to the Strengthening the Reporting of Observational Studies in Epidemiology (STROBE) guidelines for observational research (von Elm et al. 2008).

### 2.2 Data Extraction

The extracted dataset included a comprehensive distribution of demographic and epidemiological variables, including census region, sex, race, and urban–rural classification. Census regions were categorized into four geographic zones: West, Midwest, South, and Northeast. Sex was classified as male or female. Racial and ethnic groups included Non‐Hispanic (NH) White, NH Black or African American, NH others (Asian, American Indian, Alaska Native, and other minority groups), and Hispanic/Latino. Similar classification have been used in earlier studies conducted using data from the CDC WONDER database (M. Ahmed, Javaid, et al. 2024; M. Ahmed, Nofal, et al. 2024; R. Ahmed et al. 2024; Ansari et al. 2025). In addition, trends in hypertension and hemorrhagic stroke were analyzed based on county‐level urbanization classifications, with rural areas encompassing noncore and micropolitan regions, while urban areas included metropolitan regions (large, medium, and small) based on the 2013 National Center for Health Statistics Urban–Rural Classification Scheme (Ingram and Franco. 2014).

### 2.3 Statistical Analysis

Crude and age‐adjusted mortality rates (AAMRs) per 100,000 population were determined. Crude mortality rates (CMRs) were calculated by dividing the number of hemorrhagic stroke‐related deaths in individuals with hypertension by the corresponding US population for each year. AAMRs were computed using the direct method of age standardization to the 2000 US standard population to allow for accurate comparisons over time (Anderson and Rosenberg 1998). The Joinpoint Regression Program (Joinpoint V 5.2.0.0, National Cancer Institute) was used to assess trends in AAMRs and CMRs from 1999 to 2023 (National Cancer Institute n.d.). This method identifies significant temporal changes in mortality by fitting log‐linear regression models where trend variations occur. Annual percent changes (APCs) with 95% confidence intervals (CIs) were calculated for each time segment at identified join points using the Monte Carlo permutation test. APCs were considered increasing or decreasing if the slope describing the mortality trend was significantly different from zero, determined using two‐tailed *t*‐testing. Statistical significance was set at *p* < 0.05.

## 3 Results

### 3.1 Annual Trends

**FIGURE 1:**
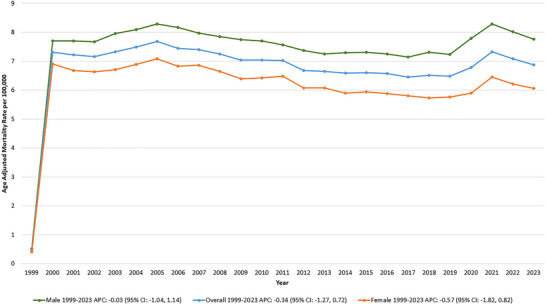
Overall and sex‐stratified hemorrhagic stroke and hypertension‐related age‐adjusted mortality rates (AAMRs) per 100,000 individuals in the United States, 1999–2023.

A total of 372,922 deaths were identified among adults (≥ 25 years) between 1999 and 2023, attributed to hemorrhagic stroke and hypertension. The AAMR was 0.45 in 1999 and 6.88 in 2023, with no significant trend observed over the study period (APC: −0.34 [95% CI: −1.27, 0.72]; *p* = 0.50) (Tables S1–S3, Figure 1).

### 3.2 Hemorrhagic Stroke and Hypertension‐Related AAMR Stratified by Sex

Throughout the study period, males consistently exhibited a higher AAMR than females. In males, the AAMR was 0.51 in 1999 and 7.76 in 2023, with no significant trend observed (APC: −0.03 [95% CI: −1.04, 1.14]; *p* = 0.97). Similarly, in females, the AAMR remained stable, ranging from 0.41 in 1999 to 6.06 in 2023, without a statistically significant trend (APC: −0.57 [95% CI: −1.82, 0.82]; *p* = 0.38) (Tables S2 and S3, Figure 1).

### 3.3 Hemorrhagic Stroke and Hypertension‐Related AAMR Stratified Race/Ethnicity

Over the study period, the highest AAMR was observed in NH Black or African American group, followed by the NH other group, the Hispanic or Latino group and lastly, the NH White populations.

For NH Black or African American individuals, the AAMR was 1.47 in 1999 and 10.95 in 2023, with a significant declining trend over time (APC: −1.68* [95% CI: −3.13, −0.14]; *p* = 0.04).

Similarly, in NH other populations, a significant decline was observed (APC: −1.65* [95% CI: −2.63, −0.40]; *p* = 0.01), with the AAMR shifting from 0.81 in 1999 to 8.2 in 2023.

**FIGURE 2:**
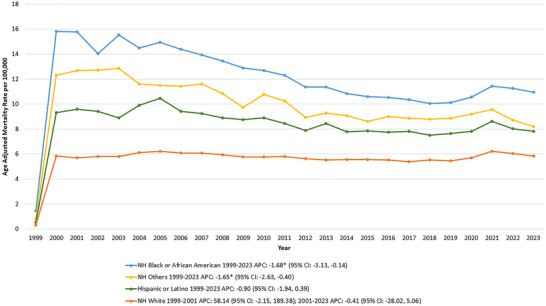
Hemorrhagic stroke and hypertension‐related age‐adjusted mortality rates (AAMRs) per 100,000 individuals stratified by race/ethnicity in the United States, 1999–2023.

In contrast, AAMR trends remained relatively stable over time for Hispanic or Latino individuals and NH White populations (Tables S2 and S4, Figure 2).

### 3.4 Hemorrhagic Stroke and Hypertension‐Related AAMR Stratified by Geographical Region

#### 3.4.1 Statewide

Between 1999 and 2020, states falling within the top 90th percentile for mortality rates included Nevada, Texas, Hawaii, California, and District of Columbia, while those in the bottom 10th percentile were Utah, Maine, Montana, Colorado, Kansas, and Massachusetts.

In the subsequent period from 2021 to 2023, the states with the highest mortality rates were Oklahoma, South Carolina, District of Columbia, Mississippi, and Nevada, whereas Maine, Connecticut, Massachusetts, Utah, and Alaska ranked in the lowest 10th percentile (Table S5).

#### 3.4.2 Census Region

Among the regions, the highest hemorrhagic stroke and hypertension‐related mortality rates were observed in the West, followed by the South, the Midwest, and lastly, the Northeast.

In the West, the AAMR was 0.50 in 1999 and 7.91 in 2023, with no significant trend over time (APC: −0.36 [95% CI: −1.46, 0.88]; *p* = 0.55). Similarly, the South showed no significant change, with AAMR ranging from 0.51 in 1999 to 7.76 in 2023 (APC: −0.01 [95% CI: −1.15, 1.30]; *p* = 0.92).

**FIGURE 3:**
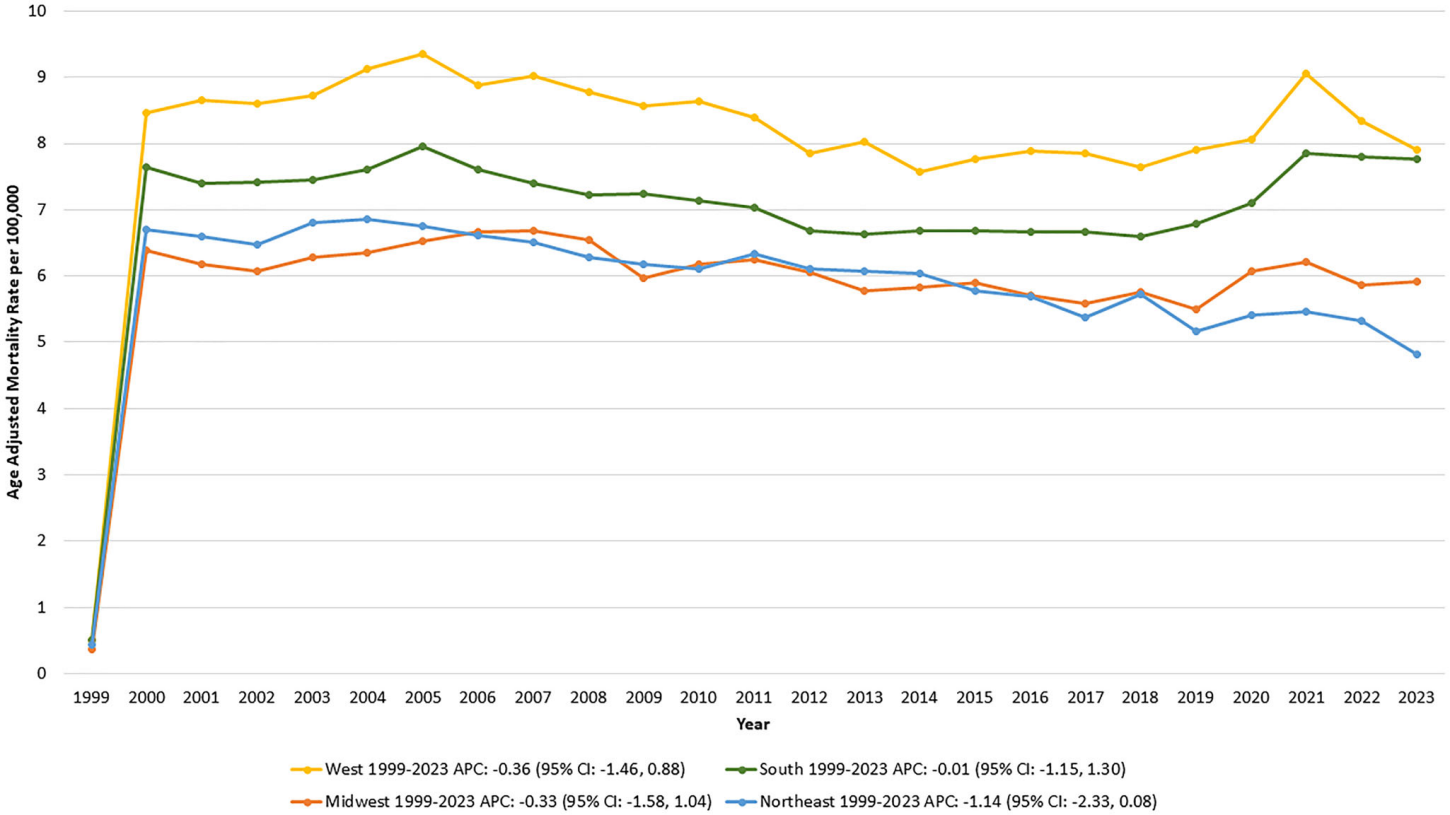
Hemorrhagic stroke and hypertension‐related age‐adjusted mortality rates (AAMRs) per 100,000 individuals stratified by census region in the United States, 1999–2023.

The Midwest also exhibited a stable trend, with an AAMR of 0.37 in 1999 and 5.91 in 2023 (APC: −0.33 [95% CI: −1.58, 1.04]; *p* = 0.59). Likewise, in the Northeast, the AAMR declined from 0.43 in 1999 to 4.82 in 2023; however, this trend did not reach statistical significance (APC: −1.14 [95% CI: −2.33, 0.08]; *p* = 0.06) (Table S6, Figure 3).

#### 3.4.3 Urban–Rural

From 1999 to 2020, urban areas displayed consistently higher hemorrhagic stroke and hypertension‐related AAMR than rural areas.

**FIGURE 4:**
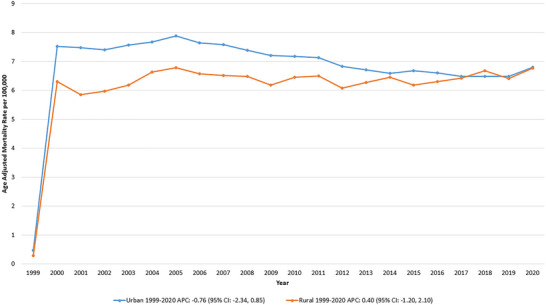
Hemorrhagic stroke and hypertension‐related age‐adjusted mortality rates (AAMRs) per 100,000 individuals stratified by urbanization in the United States, 1999–2020. Data for urbanization AAMRs was unavailable for 2021–2023.

In urban areas, the AAMR was 0.47 in 1999 and 6.80 in 2020, with no significant trend observed (APC: −0.76 [95% CI: −2.34, 0.85]; *p* = 0.32). Similarly, rural areas exhibited stable trends, with the AAMR increasing from 0.29 in 1999 to 6.78 in 2020, though this change was not statistically significant (APC: 0.40 [95% CI: −1.20, 2.10]; *p* = 0.49) (Table S7, Figure 4).

## 4 Discussion

This retrospective analysis of mortality data from the CDC WONDER database reveals several key findings. First, the overall AAMR for hemorrhagic stroke and hypertension remained stable from 1999 to 2023, with no significant trend observed. Males consistently exhibited higher AAMRs than females throughout the study period. Considerable variations in mortality were observed among racial subgroups, with NH Black or African American populations exhibiting the highest AAMR. Regional disparities were evident, with the highest mortality rates observed in the West. From 1999 to 2020, states in the top 90th percentile had more than twice the AAMR of those in the bottom 10th percentile, with this disparity becoming even more pronounced in the 2021–2023 period. Urban areas consistently had higher AAMRs than rural areas, though neither showed a significant change over time. Lastly, mortality rates increased with age, with the highest burden observed in individuals aged 85 years and older.

Despite the global rise in hypertension prevalence and related mortality, our analysis indicates that the AAMR for hemorrhagic stroke and hypertension in the United States remained stable from 1999 to 2023 (Mills et al. 2020; Forrester et al. 2020). This stability may be attributed to advancements in hypertension management, including the widespread availability of effective antihypertensive medications and improved treatment protocols, which have likely prevented further rise in mortality rates (Gorelick et al. 2020). However, recent trends indicate a decline in overall stroke‐related mortality in the United States, highlighting potential disparities in outcomes between ischemic and hemorrhagic subtypes (Ananth et al. 2023). Unlike ischemic stroke, where thrombolysis and thrombectomy have significantly reduced mortality, hemorrhagic stroke lacks similarly effective acute interventions (Park et al. 2022). However, advancements in hypertension management have likely helped stabilize its incidence and mortality by reducing the prevalence of poorly controlled blood pressure, the primary modifiable risk factor (Diener and Hankey 2020). Chronic hypertension induces structural alterations in small cerebral arteries, such as lipohyalinosis, fibrinoid necrosis, and microaneurysm formation, which weaken vessel walls and increase susceptibility to rupture, leading to ICH (Leasure et al. 2019; Hainsworth et al. 2024). In addition, sustained hypertension contributes to the formation and rupture of saccular aneurysms, increasing the risk of SAH (Greving et al. 2014). Consequently, chronic elevated blood pressure is a major risk factor for both intracerebral and SAHs, highlighting the importance of aggressive blood pressure management to lower the risk of hemorrhagic stroke.

While advances in hypertension treatment have mitigated mortality risks, they have not been sufficient to reduce hemorrhagic stroke burden. Poor prognosis and limited treatment options further compound this challenge, especially as many individuals remain undiagnosed, untreated, or inadequately managed. A cross‐sectional study revealed that over half of US adults with uncontrolled hypertension were unaware of their condition and remained untreated, while 70.8% of those receiving treatment still had inadequately controlled blood pressure (Richardson et al. 2024). In addition to systemic gaps in prevention and management, several radiological and clinical factors have been identified as a strong, independent determinant of poor early outcomes. In a registry‐based study of patients with thalamic hemorrhage, the presence of intraventricular hemorrhage (IVH) significantly increased the risk of in‐hospital mortality, along with altered consciousness and older age (Arboix et al. 2007). IVH may lead to obstructive hydrocephalus, elevated intracranial pressure, and greater perihematomal injury, all of which complicate early management and worsen prognosis. These complications highlight why hemorrhagic stroke continues to have disproportionately poor outcomes despite medical advances.

Our findings on sex disparities align with existing literature indicating men have higher risk for hemorrhagic stroke than women (Ashraf et al. 2025; Barker‐Collo et al. 2015). Specifically, ICH occurs more frequently in men while SAH disproportionately affects women (Osteraas 2023; Kalasapudi et al. 2023). This disparity may be partly explained by differences in traditional risk factors such as higher rates of hypertension, smoking, and dyslipidemia among men, which are more strongly associated with ICH (Appelros et al. 2009; Ahangar et al. 2018). In addition, hormonal influences may play a role in these differences, as estrogen's neuroprotective effects may help reduce stroke risk in premenopausal women while late menopause and estrogen deficiency are linked to increased subarachnoid hemorrhagic stroke risk (Fuentes et al. 2022; Poorthuis et al. 2017). Although stroke incidence increases with age in both sexes, men tend to experience their first stroke at a younger age than women. Moreover, while men have higher age‐specific stroke rates, women experience a greater overall stroke burden due to their longer life expectancy. Women also tend to experience more severe strokes, with one study reporting a 1‐month case fatality rate of 24.7% in women compared to 19.7% in men (Appelros et al. 2009). Despite this, they tend to survive longer but often experience greater disability, potentially due to their later stroke onset compared to men (Appelros et al. 2009; Ali et al. 2022). This may be further influenced by disparities in acute treatment and rehabilitation access, as women are less likely to receive intensive care interventions such as intubation and fever management, which may contribute to worse functional outcomes (Carcel et al. 2019).

We found significant racial/ethnic disparities in hemorrhagic stroke and hypertension‐related mortality, with the highest rates observed among NH Black or African American individuals. These findings are consistent with the existing literature that indicates higher prevalence of hypertension and lower blood pressure control rates in this population (Aggarwal et al. 2021). The earlier onset of hypertension in African American individuals suggests a potential genetic predisposition, while disparities in healthcare access, utilization, and quality may contribute to lower blood pressure control despite higher awareness and treatment rates (Aggarwal et al. 2021; Whelton et al. 2016). The past decade has seen a decline in BP control rates, with NH Black individuals having significantly lower control rates than NH White counterparts, further exacerbating these disparities (Abrahamowicz et al. 2023). This uncontrolled hypertension contributes to the increased recurrence of ICH and worse 30‐day survival rates in NH Black patients following ICH, likely due to disparities in acute and post‐acute stroke care, access to specialized interventions, and timely management (Rodriguez‐Torres et al. 2018; Tarko et al. 2022). Racial and ethnic disparities extend beyond acute stroke care, as NH Black and Hispanic patients are less likely to be discharged to facilities providing intensive post‐acute care, which may further worsen long‐term outcomes and recovery rates (di Lorenzo et al. 2023). In contrast, NH White populations exhibit lower AAMR for hemorrhagic stroke and hypertension‐related mortality, likely due to better BP control, greater treatment adherence, and improved healthcare access. Despite lower hypertension treatment rates, NH Whites achieve better control, possibly due to higher education levels and lower BMI, contributing to their comparatively favorable outcomes (Vu et al. 2020).

While Hispanics and Asians have similar prevalence rates to NH White individuals, they face challenges in awareness and treatment (Aggarwal et al. 2021; Opara et al. 2013). The Hispanic Community Health Study/Study of Latinos reported a hypertension prevalence of 25.5%, with control rates at 37.5%, significantly lower than the 56.3% control rate observed in NH Whites (Sorlie et al. 2014). Factors such as language barriers, cultural differences, and limited healthcare access may contribute to these disparities. However, studies like the Antihypertensive and Lipid‐Lowering Treatment to Prevent Heart Attack Trial (ALLHAT) have demonstrated that, in controlled settings with equal access to care and medications, Hispanic participants achieved BP control rates comparable to or better than non‐Hispanic participants (Margolis et al. 2007). This suggests that, when provided equitable healthcare resources, Hispanic individuals can attain effective BP management. In addition, Hispanic patients may experience disparities in long‐term BP management after ICH, which could contribute to higher recurrence rates (Rodriguez‐Torres et al. 2018). Hispanic patients are also at a greater risk of 30‐day mortality after SAH compared to NH White patients, emphasizing the need for improved acute stroke care and post‐discharge management in this population (Tarko et al. 2022). These disparities extend to post‐acute care, as Hispanics have the lowest rate of facility discharge, potentially limiting access to rehabilitation services and further impacting recovery outcomes (di Lorenzo et al. 2023).

We observed higher mortality rates in urban areas than in rural areas, with the difference decreasing over time. This disparity may be linked to factors more prevalent in urban settings, including higher rates of hypertension, diabetes, smoking, greater comorbidity burden, and less physically active lifestyles, all of which increase the risk of hemorrhagic stroke (Estes et al. 2009). However, while our study found higher mortality in urban areas, existing literature often reports a greater prevalence of hypertension and stroke risk factors in rural regions (Kamin Mukaz et al. 2022). This discrepancy may stem from limited healthcare access in rural areas, where individuals are more likely to remain undiagnosed and underreported due to fewer medical facilities and reduced healthcare utilization (Kamin Mukaz et al. 2022). Therefore, our findings should be interpreted with caution, considering these differences. In addition, we found that the West region had the highest mortality, which may be linked to regional disparities in hypertension prevalence, comorbid conditions such as obesity and diabetes, and healthcare access limitations affecting stroke outcomes. A shortage of medical facilities in certain areas of the western United States has been associated with higher rates of hypertension‐related complications, including more severe cases and increased mortality (Raja et al. 2023). Future research should aim to identify the underlying factors driving these variations to improve outcomes.

Some limitations must be acknowledged when interpreting our findings. Our reliance on the CDC WONDER database means that cause‐of‐death classification is based on death certificates, which may introduce inaccuracies. Misclassification of hemorrhagic stroke deaths due to ICD coding differences remains a concern, particularly when differentiating between subtypes of stroke or attributing deaths to underlying hypertension. In addition, the absence of clinical details, such as stroke severity, comorbidity burden, or treatment history (e.g., antihypertensive or anticoagulant use), limits our ability to contextualize individual mortality outcomes. The database also does not distinguish between hemorrhagic stroke subtypes based on anatomical location (e.g., lobar vs. deep subcortical hemorrhages). This is a notable limitation, as lobar hemorrhages often result from non‐hypertensive mechanisms such as cerebral amyloid angiopathy and may present a different clinical course and a more severe early prognosis compared to deep subcortical hemorrhages (Mendiola et al. 2023). Furthermore, the database lacks individual‐level factors such as socioeconomic status, healthcare access, and lifestyle behaviors, all of which play significant roles in stroke risk and outcomes. While age adjustment was performed, it does not fully account for evolving trends in hypertension management or broader healthcare improvements over time.

Nonetheless, our study has several strengths. By addressing a significant gap in the literature, it provides a comprehensive analysis of hemorrhagic stroke and hypertension‐related mortality trends in the United States. Our findings emphasize the significant impact of hypertension on mortality patterns and reveal enduring geographic disparities, especially in regions with restricted healthcare access. In addition, the use of a large, nationally representative dataset strengthens the generalizability of our results.

## 5 Conclusion

Over the study period, hemorrhagic stroke and hypertension‐related mortality remained stable, with notable disparities across demographic and geographic groups. Males, NH Black or African American individuals, and older adults experienced the highest mortality burden. Regionally, the West had the highest mortality rates, while urban areas consistently exhibited greater mortality than rural areas, though this gap has narrowed over time. These findings highlight the need for improved hypertension management, targeted interventions for high‐risk populations, and equitable healthcare access to reduce disparities and improve outcomes. Future research should focus on integrating clinical stroke registries with mortality data to investigate how factors such as hemorrhage location, stroke severity, blood pressure control, and treatment history influence outcomes.

## Author Contributions

**Muhammed Ameen Noushad**: writing – original draft, methodology, formal analysis. **Emer Mulholland**: writing – original draft, investigation, software, formal analysis. **Jaisurya Jaisukhalal**: writing – original draft, software, formal analysis. **Reem Abukhater**: writing – original draft, data curation. **Wahhaj Qayyum**: writing – original draft, data curation. **Munikaverappa Anjanappa Mukesh**: investigation, data curation. **Jovita Jerome D silva**: writing – original draft, methodology. **Mubashir Tanvir Sheikh**: writing – original draft, methodology, formal analysis. **Muhammad Muneeb Arshad**: writing – original draft, project administration. **Naweed Ebrahim Essa**: writing – original draft. **Eeshal Zulfiqar**: writing – review and editing, validation, visualization, supervision. **Mushood Ahmed**: writing – review and editing, supervision, visualization. **Raheel Ahmed**: validation, writing – review and editing, supervision, project administration.

## Ethics Statement

The authors have nothing to report.

## Consent

The authors have nothing to report.

## Conflicts of Interest

The authors declare no conflicts of interest.

## Peer Review

The peer review history for this article is available at https://publons.com/publon/10.1002/brb3.70704

## Supporting information



Supplementary Appendix

## Data Availability

All data generated or analyzed during this study are included in this article. Further inquiries can be directed to the corresponding author.
