# Supplementary material for: Trends and Disparities in Hemorrhagic Stroke and Hypertension‐Related Mortality in the United States From 1999 to 2023: A CDC WONDER Database Analysis
Source: Brain Behav. 2025 Jul 21;15(7):e70704. doi: 10.1002/brb3.70704 (PMC12277664; doi:10.1002/brb3.70704)
Supplement: Supplementary file 1 — Supplementary Appendix [file BRB3-15-e70704-s001.docx]

**Supplementary Appendix**

**Supplemental Table 1: Hemorrhagic stroke and hypertension-related deaths, Stratified by Sex and Race in the United States, 1999 to 2023**

| Year | Overall | Female | Male | NH Black or African American | NH White | Hispanic or Latino | NH Others |
| --- | --- | --- | --- | --- | --- | --- | --- |
| 1999 | 808 | 413 | 395 | 248 | 444 | 59 | 44 |
| 2000 | 13100 | 7290 | 5810 | 2666 | 8745 | 980 | 670 |
| 2001 | 13134 | 7168 | 5966 | 2729 | 8586 | 1055 | 728 |
| 2002 | 13259 | 7210 | 6049 | 2506 | 8854 | 1078 | 779 |
| 2003 | 13808 | 7401 | 6407 | 2819 | 9006 | 1098 | 850 |
| 2004 | 14338 | 7703 | 6635 | 2703 | 9541 | 1271 | 790 |
| 2005 | 14995 | 8057 | 6938 | 2860 | 9861 | 1422 | 827 |
| 2006 | 14846 | 7874 | 6972 | 2831 | 9775 | 1350 | 859 |
| 2007 | 15017 | 8048 | 6969 | 2778 | 9881 | 1411 | 924 |
| 2008 | 14937 | 7927 | 7010 | 2785 | 9828 | 1392 | 908 |
| 2009 | 14805 | 7757 | 7048 | 2721 | 9715 | 1467 | 855 |
| 2010 | 15064 | 7899 | 7165 | 2714 | 9796 | 1522 | 981 |
| 2011 | 15350 | 8121 | 7229 | 2697 | 10068 | 1537 | 1009 |
| 2012 | 14966 | 7799 | 7167 | 2567 | 9921 | 1507 | 922 |
| 2013 | 15172 | 7918 | 7254 | 2598 | 9852 | 1674 | 1003 |
| 2014 | 15316 | 7856 | 7460 | 2561 | 9994 | 1654 | 1058 |
| 2015 | 15607 | 7992 | 7615 | 2569 | 10137 | 1764 | 1065 |
| 2016 | 15859 | 8126 | 7733 | 2599 | 10232 | 1804 | 1158 |
| 2017 | 15985 | 8201 | 7784 | 2638 | 10146 | 1935 | 1203 |
| 2018 | 16391 | 8220 | 8171 | 2608 | 10592 | 1897 | 1247 |
| 2019 | 16631 | 8413 | 8218 | 2659 | 10581 | 2026 | 1327 |
| 2020 | 17685 | 8693 | 8992 | 2854 | 11192 | 2178 | 1430 |
| 2021 | 18748 | 9205 | 9543 | 3034 | 11664 | 2449 | 1437 |
| 2022 | 18754 | 9283 | 9471 | 3029 | 11774 | 2378 | 1399 |
| 2023 | 18347 | 9070 | 9277 | 2994 | 11448 | 2379 | 1359 |

NH, Non-Hispanic

**Supplemental Table 2: Annual percent change (APC) of Hemorrhagic stroke and hypertension-related age-adjusted mortality rates per 100,000 in the United States, 1999 to 2023**

| Year Interval | APC (95% confidence interval) |
| --- | --- |
| **Overall** |  |
| 1999-2023 | -0.34 (-1.27, 0.72) |
| **Sex** |  |
| **Female** |  |
| 1999-2023 | -0.57 (-1.82, 0.82) |
| **Male** |  |
| 1999-2023 | -0.03 (-1.04, 1.14) |
| **Race** |  |
| **NH Black or African American** |  |
| 1999-2023 | -1.68* (-3.13, -0.14) |
| **NH White** |  |
| 1999-2001 | 58.14 (-2.15, 189.38) |
| 2001-2023 | -0.41 (-28.02, 5.06) |
| **Hispanic or Latino** |  |
| 1999-2023 | -0.90 (-1.94, 0.39) |
| **NH Others** |  |
| 1999-2023 | -1.65* (-2.63, -0.40) |
| **Census region** |  |
| **Northeast** |  |
| 1999-2023 | -1.14 (-2.33, 0.08) |
| **Midwest** |  |
| 1999-2023 | -0.33 (-1.58, 1.04) |
| **South** |  |
| 1999-2023 | -0.01 (-1.15, 1.30) |
| **West** |  |
| 1999-2023 | -0.36 (-1.46, 0.88) |
| **Urbanization** |  |
| **Urban** |  |
| 1999-2020 | -0.76 (-2.34, 0.85) |
| **Rural** |  |
| 1999-2020 | 0.40 (-1.20, 2.10) |
| **Ten-year Age groups**** |  |
| **35-44 years** |  |
| 1999-2001 | 45.40 (-0.58, 107.06) |
| 2001-2023 | 0.43 (-8.83, 5.31) |
| **45-54 years** |  |
| 1999-2023 | -0.18 (-1.67, 1.36) |
| **55-64 years** |  |
| 1999-2023 | -0.52 (-1.59, 0.70) |
| **65-74 years** |  |
| 1999-2023 | -1.16 (-2.28, 0.16) |
| **75-84 years** |  |
| 1999-2023 | -0.64 (-1.85, 0.69) |
| **85+ years** |  |
| 1999-2023 | 0.72 (-0.36, 2.01) |

* Significant values

** For age groups, crude mortality rates were used for calculating APCs

**Supplemental Table 3: Overall and Sex‐Stratified** **Hemorrhagic stroke and hypertension-related Age-Adjusted Mortality Rates per 100,000 in the United States, 1999 to 2023**

|  | Age-Adjusted Rate (95% confidence interval) | | |
| --- | --- | --- | --- |
| Year | **Overall** | **Female** | **Male** |
| 1999 | 0.45 (0.42 - 0.48) | 0.41 (0.37 - 0.45) | 0.51 (0.46 - 0.56) |
| 2000 | 7.31 (7.19 - 7.44) | 6.91 (6.75 - 7.07) | 7.7 (7.49 - 7.9) |
| 2001 | 7.23 (7.11 - 7.36) | 6.69 (6.54 - 6.85) | 7.71 (7.51 - 7.91) |
| 2002 | 7.16 (7.04 - 7.28) | 6.63 (6.47 - 6.78) | 7.67 (7.47 - 7.87) |
| 2003 | 7.33 (7.2 - 7.45) | 6.72 (6.57 - 6.87) | 7.96 (7.77 - 8.16) |
| 2004 | 7.49 (7.36 - 7.61) | 6.9 (6.75 - 7.06) | 8.1 (7.9 - 8.29) |
| 2005 | 7.69 (7.57 - 7.82) | 7.09 (6.93 - 7.25) | 8.29 (8.09 - 8.49) |
| 2006 | 7.45 (7.33 - 7.57) | 6.83 (6.68 - 6.99) | 8.17 (7.98 - 8.37) |
| 2007 | 7.4 (7.28 - 7.52) | 6.87 (6.72 - 7.03) | 7.97 (7.78 - 8.16) |
| 2008 | 7.25 (7.13 - 7.36) | 6.65 (6.5 - 6.8) | 7.85 (7.67 - 8.04) |
| 2009 | 7.04 (6.92 - 7.15) | 6.39 (6.25 - 6.53) | 7.75 (7.56 - 7.93) |
| 2010 | 7.05 (6.93 - 7.16) | 6.43 (6.29 - 6.57) | 7.7 (7.52 - 7.88) |
| 2011 | 7.03 (6.92 - 7.15) | 6.48 (6.34 - 6.63) | 7.57 (7.4 - 7.75) |
| 2012 | 6.69 (6.58 - 6.79) | 6.08 (5.94 - 6.22) | 7.38 (7.2 - 7.55) |
| 2013 | 6.65 (6.54 - 6.76) | 6.08 (5.94 - 6.21) | 7.26 (7.09 - 7.43) |
| 2014 | 6.59 (6.48 - 6.69) | 5.9 (5.76 - 6.03) | 7.3 (7.13 - 7.47) |
| 2015 | 6.6 (6.5 - 6.71) | 5.94 (5.81 - 6.07) | 7.32 (7.16 - 7.49) |
| 2016 | 6.57 (6.47 - 6.67) | 5.88 (5.75 - 6.01) | 7.25 (7.09 - 7.42) |
| 2017 | 6.45 (6.35 - 6.55) | 5.81 (5.68 - 5.94) | 7.15 (6.99 - 7.31) |
| 2018 | 6.51 (6.41 - 6.61) | 5.74 (5.62 - 5.87) | 7.31 (7.14 - 7.47) |
| 2019 | 6.48 (6.38 - 6.58) | 5.77 (5.64 - 5.89) | 7.24 (7.08 - 7.4) |
| 2020 | 6.79 (6.69 - 6.89) | 5.9 (5.77 - 6.03) | 7.79 (7.63 - 7.96) |
| 2021 | 7.33 (7.22 - 7.43) | 6.45 (6.31 - 6.58) | 8.29 (8.12 - 8.46) |
| 2022 | 7.09 (6.99 - 7.19) | 6.21 (6.08 - 6.34) | 8.02 (7.86 - 8.19) |
| 2023 | 6.88 (6.78 - 6.98) | 6.06 (5.93 - 6.19) | 7.76 (7.6 - 7.92) |

**Supplemental Table 4: Hemorrhagic stroke and hypertension-related Age-Adjusted Mortality Rates per 100,000, Stratified by Race in the United States, 1999 to 2023**

|  | Age-Adjusted Rate (95% confidence interval) | | | |
| --- | --- | --- | --- | --- |
| Year | **NH Black or  African American** | **NH White** | **Hispanic or  Latino** | **NH others** |
| 1999 | 1.47 (1.28 - 1.65) | 0.31 (0.28 - 0.34) | 0.52 (0.38 - 0.68) | 0.81 (0.57 - 1.11) |
| 2000 | 15.8 (15.19 - 16.41) | 5.86 (5.73 - 5.98) | 9.31 (8.69 - 9.93) | 12.29 (11.31 - 13.26) |
| 2001 | 15.77 (15.17 - 16.38) | 5.69 (5.57 - 5.81) | 9.6 (8.98 - 10.21) | 12.69 (11.72 - 13.66) |
| 2002 | 14.04 (13.48 - 14.6) | 5.8 (5.68 - 5.92) | 9.41 (8.81 - 10.01) | 12.73 (11.79 - 13.67) |
| 2003 | 15.53 (14.94 - 16.11) | 5.82 (5.7 - 5.94) | 8.9 (8.34 - 9.46) | 12.87 (11.96 - 13.78) |
| 2004 | 14.5 (13.94 - 15.06) | 6.12 (6 - 6.25) | 9.9 (9.32 - 10.48) | 11.61 (10.76 - 12.46) |
| 2005 | 14.93 (14.37 - 15.49) | 6.21 (6.09 - 6.34) | 10.46 (9.88 - 11.04) | 11.49 (10.67 - 12.31) |
| 2006 | 14.39 (13.85 - 14.94) | 6.08 (5.96 - 6.21) | 9.42 (8.89 - 9.96) | 11.43 (10.63 - 12.22) |
| 2007 | 13.94 (13.41 - 14.48) | 6.07 (5.95 - 6.19) | 9.26 (8.75 - 9.77) | 11.6 (10.82 - 12.38) |
| 2008 | 13.44 (12.92 - 13.95) | 5.93 (5.81 - 6.05) | 8.9 (8.4 - 9.39) | 10.83 (10.1 - 11.56) |
| 2009 | 12.91 (12.41 - 13.41) | 5.78 (5.66 - 5.89) | 8.76 (8.28 - 9.24) | 9.74 (9.06 - 10.42) |
| 2010 | 12.69 (12.2 - 13.18) | 5.79 (5.67 - 5.91) | 8.89 (8.41 - 9.36) | 10.78 (10.08 - 11.48) |
| 2011 | 12.31 (11.83 - 12.79) | 5.82 (5.7 - 5.93) | 8.46 (8.01 - 8.9) | 10.26 (9.61 - 10.92) |
| 2012 | 11.35 (10.9 - 11.8) | 5.64 (5.53 - 5.75) | 7.89 (7.47 - 8.31) | 8.95 (8.35 - 9.54) |
| 2013 | 11.35 (10.9 - 11.8) | 5.54 (5.43 - 5.65) | 8.46 (8.03 - 8.88) | 9.28 (8.69 - 9.87) |
| 2014 | 10.86 (10.43 - 11.29) | 5.56 (5.45 - 5.67) | 7.79 (7.4 - 8.18) | 9.08 (8.52 - 9.64) |
| 2015 | 10.61 (10.18 - 11.03) | 5.56 (5.45 - 5.67) | 7.85 (7.47 - 8.24) | 8.61 (8.08 - 9.14) |
| 2016 | 10.52 (10.1 - 10.93) | 5.52 (5.41 - 5.63) | 7.76 (7.38 - 8.13) | 9.01 (8.48 - 9.54) |
| 2017 | 10.35 (9.94 - 10.75) | 5.38 (5.27 - 5.49) | 7.83 (7.46 - 8.19) | 8.86 (8.35 - 9.37) |
| 2018 | 10.06 (9.66 - 10.46) | 5.54 (5.43 - 5.65) | 7.5 (7.15 - 7.85) | 8.81 (8.31 - 9.31) |
| 2019 | 10.13 (9.74 - 10.53) | 5.46 (5.36 - 5.57) | 7.64 (7.29 - 7.98) | 8.87 (8.38 - 9.35) |
| 2020 | 10.55 (10.16 - 10.95) | 5.72 (5.61 - 5.83) | 7.82 (7.48 - 8.16) | 9.2 (8.71 - 9.68) |
| 2021 | 11.42 (11 - 11.84) | 6.22 (6.11 - 6.34) | 8.62 (8.26 - 8.98) | 9.57 (9.07 - 10.07) |
| 2022 | 11.25 (10.83 - 11.66) | 6.04 (5.92 - 6.15) | 8.03 (7.69 - 8.36) | 8.73 (8.27 - 9.19) |
| 2023 | 10.95 (10.55 - 11.36) | 5.86 (5.75 - 5.97) | 7.83 (7.5 - 8.15) | 8.2 (7.76 - 8.64) |

**Supplemental Table 5: Hemorrhagic stroke and hypertension-related Age-Adjusted Mortality Rates per 100,000, Stratified by States in the United States, 1999 to 2023**

| State | Age-Adjusted Rate (95% confidence interval) | |
| --- | --- | --- |
|  | **1999-2020** | **2021-2023** |
| Alabama | 6.47 (6.29 - 6.66) | 6.65 (6.17 - 7.12) |
| Alaska | 5.48 (4.89 - 6.07) | 4.71 (3.59 - 6.08) |
| Arizona | 6.38 (6.22 - 6.54) | 6.22 (5.85 - 6.6) |
| Arkansas | 6.34 (6.11 - 6.57) | 7.86 (7.2 - 8.52) |
| California | 9.8 (9.72 - 9.89) | 9.65 (9.44 - 9.86) |
| Colorado | 4.69 (4.52 - 4.86) | 6.21 (5.76 - 6.65) |
| Connecticut | 4.94 (4.77 - 5.12) | 4.18 (3.76 - 4.6) |
| Delaware | 5.68 (5.29 - 6.07) | 8.71 (7.57 - 9.85) |
| District of Columbia | 13.69 (12.9 - 14.47) | 11.43 (9.58 - 13.29) |
| Florida | 5.55 (5.47 - 5.63) | 6.32 (6.12 - 6.52) |
| Georgia | 6.52 (6.38 - 6.66) | 7.21 (6.85 - 7.56) |
| Hawaii | 9.11 (8.71 - 9.51) | 8 (7.04 - 8.97) |
| Idaho | 5.14 (4.84 - 5.44) | 7.29 (6.47 - 8.11) |
| Illinois | 5.61 (5.51 - 5.72) | 4.73 (4.48 - 4.98) |
| Indiana | 5.96 (5.81 - 6.11) | 6.59 (6.18 - 7) |
| Iowa | 5.34 (5.15 - 5.54) | 6.54 (5.97 - 7.11) |
| Kansas | 4.75 (4.54 - 4.95) | 6.63 (6.01 - 7.26) |
| Kentucky | 5.91 (5.72 - 6.1) | 7.24 (6.72 - 7.76) |
| Louisiana | 7.03 (6.83 - 7.23) | 7.09 (6.56 - 7.61) |
| Maine | 4.15 (3.89 - 4.41) | 2.13 (1.7 - 2.63) |
| Maryland | 7.83 (7.64 - 8.02) | 9.7 (9.19 - 10.21) |
| Massachusetts | 4.78 (4.65 - 4.91) | 4.28 (3.97 - 4.59) |
| Michigan | 6.43 (6.31 - 6.56) | 6.13 (5.81 - 6.45) |
| Minnesota | 5.75 (5.58 - 5.91) | 7.25 (6.79 - 7.71) |
| Mississippi | 7.67 (7.41 - 7.93) | 12.28 (11.43 - 13.14) |
| Missouri | 5.31 (5.16 - 5.45) | 5.24 (4.86 - 5.61) |
| Montana | 4.33 (4.02 - 4.65) | 5.69 (4.77 - 6.61) |
| Nebraska | 6.15 (5.87 - 6.43) | 8.25 (7.39 - 9.1) |
| Nevada | 7.86 (7.57 - 8.15) | 12.32 (11.48 - 13.15) |
| New Hampshire | 5.24 (4.93 - 5.55) | 5.46 (4.69 - 6.23) |
| New Jersey | 5.95 (5.82 - 6.08) | 5.02 (4.72 - 5.31) |
| New Mexico | 5.64 (5.37 - 5.91) | 6.03 (5.34 - 6.72) |
| New York | 7.09 (6.99 - 7.18) | 5.78 (5.56 - 6) |
| North Carolina | 6.81 (6.67 - 6.94) | 6.02 (5.71 - 6.33) |
| North Dakota | 5.77 (5.34 - 6.21) | 6.31 (5.1 - 7.52) |
| Ohio | 6.53 (6.41 - 6.64) | 5.82 (5.54 - 6.1) |
| Oklahoma | 6.99 (6.77 - 7.2) | 10.44 (9.75 - 11.12) |
| Oregon | 6.23 (6.03 - 6.42) | 7.73 (7.19 - 8.27) |
| Pennsylvania | 5.12 (5.03 - 5.21) | 5.39 (5.14 - 5.64) |
| Rhode Island | 7.25 (6.86 - 7.64) | 6.97 (6 - 7.94) |
| South Carolina | 7.36 (7.16 - 7.56) | 11.3 (10.7 - 11.9) |
| South Dakota | 5.29 (4.91 - 5.68) | 7.95 (6.74 - 9.17) |
| Tennessee | 7.34 (7.17 - 7.51) | 8.81 (8.34 - 9.28) |
| Texas | 8.7 (8.6 - 8.8) | 9.56 (9.3 - 9.82) |
| Utah | 3.96 (3.74 - 4.19) | 4.52 (3.97 - 5.08) |
| Vermont | 7.41 (6.89 - 7.93) | 7.17 (5.91 - 8.42) |
| Virginia | 4.87 (4.74 - 4.99) | 4.76 (4.45 - 5.06) |
| Washington | 7.35 (7.18 - 7.52) | 8.58 (8.14 - 9.02) |
| West Virginia | 6.82 (6.54 - 7.11) | 5.64 (4.95 - 6.33) |
| Wisconsin | 5.14 (5 - 5.29) | 5.98 (5.57 - 6.38) |
| Wyoming | 4.95 (4.46 - 5.43) | 6.48 (5.18 - 8) |

**Supplemental Table 6: Hemorrhagic stroke and hypertension-related Age-Adjusted Mortality Rates per 100,000, Stratified by Census Region in the United States, 1999 to 2023**

| Census region | Year | Age-Adjusted Rate (95% confidence interval) |
| --- | --- | --- |
| **Northeast** |  |  |
| Northeast | 1999 | 0.43 (0.36 - 0.5) |
| Northeast | 2000 | 6.7 (6.43 - 6.96) |
| Northeast | 2001 | 6.59 (6.33 - 6.85) |
| Northeast | 2002 | 6.47 (6.22 - 6.73) |
| Northeast | 2003 | 6.8 (6.54 - 7.06) |
| Northeast | 2004 | 6.86 (6.6 - 7.12) |
| Northeast | 2005 | 6.76 (6.5 - 7.01) |
| Northeast | 2006 | 6.62 (6.37 - 6.88) |
| Northeast | 2007 | 6.5 (6.25 - 6.74) |
| Northeast | 2008 | 6.28 (6.04 - 6.53) |
| Northeast | 2009 | 6.18 (5.94 - 6.42) |
| Northeast | 2010 | 6.1 (5.86 - 6.33) |
| Northeast | 2011 | 6.33 (6.09 - 6.58) |
| Northeast | 2012 | 6.11 (5.87 - 6.34) |
| Northeast | 2013 | 6.08 (5.85 - 6.31) |
| Northeast | 2014 | 6.03 (5.8 - 6.26) |
| Northeast | 2015 | 5.77 (5.54 - 6) |
| Northeast | 2016 | 5.68 (5.46 - 5.9) |
| Northeast | 2017 | 5.38 (5.17 - 5.6) |
| Northeast | 2018 | 5.72 (5.5 - 5.94) |
| Northeast | 2019 | 5.16 (4.96 - 5.37) |
| Northeast | 2020 | 5.41 (5.19 - 5.62) |
| Northeast | 2021 | 5.47 (5.26 - 5.68) |
| Northeast | 2022 | 5.33 (5.13 - 5.54) |
| Northeast | 2023 | 4.82 (4.62 - 5.02) |
| **Midwest** |  |  |
| Midwest | 1999 | 0.37 (0.31 - 0.43) |
| Midwest | 2000 | 6.38 (6.14 - 6.62) |
| Midwest | 2001 | 6.17 (5.93 - 6.4) |
| Midwest | 2002 | 6.07 (5.84 - 6.3) |
| Midwest | 2003 | 6.29 (6.06 - 6.53) |
| Midwest | 2004 | 6.35 (6.12 - 6.59) |
| Midwest | 2005 | 6.52 (6.28 - 6.75) |
| Midwest | 2006 | 6.66 (6.42 - 6.9) |
| Midwest | 2007 | 6.68 (6.45 - 6.92) |
| Midwest | 2008 | 6.54 (6.31 - 6.77) |
| Midwest | 2009 | 5.97 (5.75 - 6.19) |
| Midwest | 2010 | 6.17 (5.95 - 6.39) |
| Midwest | 2011 | 6.24 (6.02 - 6.47) |
| Midwest | 2012 | 6.06 (5.84 - 6.28) |
| Midwest | 2013 | 5.78 (5.57 - 5.99) |
| Midwest | 2014 | 5.83 (5.62 - 6.05) |
| Midwest | 2015 | 5.89 (5.68 - 6.1) |
| Midwest | 2016 | 5.71 (5.5 - 5.91) |
| Midwest | 2017 | 5.58 (5.37 - 5.78) |
| Midwest | 2018 | 5.76 (5.55 - 5.96) |
| Midwest | 2019 | 5.49 (5.29 - 5.69) |
| Midwest | 2020 | 6.07 (5.86 - 6.28) |
| Midwest | 2021 | 6.21 (6 - 6.43) |
| Midwest | 2022 | 5.86 (5.65 - 6.06) |
| Midwest | 2023 | 5.91 (5.7 - 6.12) |
| **South** |  |  |
| South | 1999 | 0.51 (0.45 - 0.57) |
| South | 2000 | 7.64 (7.42 - 7.86) |
| South | 2001 | 7.4 (7.19 - 7.61) |
| South | 2002 | 7.42 (7.21 - 7.63) |
| South | 2003 | 7.45 (7.24 - 7.65) |
| South | 2004 | 7.61 (7.41 - 7.82) |
| South | 2005 | 7.95 (7.74 - 8.16) |
| South | 2006 | 7.6 (7.4 - 7.8) |
| South | 2007 | 7.4 (7.2 - 7.6) |
| South | 2008 | 7.23 (7.04 - 7.43) |
| South | 2009 | 7.24 (7.05 - 7.44) |
| South | 2010 | 7.13 (6.94 - 7.32) |
| South | 2011 | 7.03 (6.85 - 7.22) |
| South | 2012 | 6.68 (6.5 - 6.86) |
| South | 2013 | 6.63 (6.45 - 6.8) |
| South | 2014 | 6.68 (6.5 - 6.85) |
| South | 2015 | 6.68 (6.51 - 6.86) |
| South | 2016 | 6.67 (6.5 - 6.84) |
| South | 2017 | 6.66 (6.49 - 6.83) |
| South | 2018 | 6.6 (6.43 - 6.77) |
| South | 2019 | 6.79 (6.63 - 6.96) |
| South | 2020 | 7.1 (6.93 - 7.27) |
| South | 2021 | 7.86 (7.68 - 8.04) |
| South | 2022 | 7.8 (7.62 - 7.97) |
| South | 2023 | 7.76 (7.58 - 7.93) |
| **West** |  |  |
| West | 1999 | 0.5 (0.42 - 0.57) |
| West | 2000 | 8.46 (8.16 - 8.76) |
| West | 2001 | 8.66 (8.36 - 8.96) |
| West | 2002 | 8.61 (8.31 - 8.9) |
| West | 2003 | 8.73 (8.43 - 9.02) |
| West | 2004 | 9.12 (8.82 - 9.42) |
| West | 2005 | 9.36 (9.06 - 9.66) |
| West | 2006 | 8.88 (8.59 - 9.17) |
| West | 2007 | 9.03 (8.75 - 9.32) |
| West | 2008 | 8.78 (8.5 - 9.06) |
| West | 2009 | 8.56 (8.29 - 8.83) |
| West | 2010 | 8.63 (8.36 - 8.9) |
| West | 2011 | 8.4 (8.14 - 8.66) |
| West | 2012 | 7.85 (7.59 - 8.1) |
| West | 2013 | 8.03 (7.78 - 8.29) |
| West | 2014 | 7.57 (7.33 - 7.82) |
| West | 2015 | 7.77 (7.53 - 8.01) |
| West | 2016 | 7.89 (7.65 - 8.13) |
| West | 2017 | 7.85 (7.62 - 8.09) |
| West | 2018 | 7.65 (7.42 - 7.88) |
| West | 2019 | 7.9 (7.67 - 8.13) |
| West | 2020 | 8.06 (7.83 - 8.29) |
| West | 2021 | 9.06 (8.81 - 9.31) |
| West | 2022 | 8.34 (8.11 - 8.58) |
| West | 2023 | 7.91 (7.68 - 8.14) |

**Supplemental Table 7: Hemorrhagic stroke and hypertension-related Age-Adjusted Mortality Rates per 100,000 in United States stratified by Urban-Rural Classification, 1999-2020**

|  | Age-Adjusted Rate (95% confidence interval) | |
| --- | --- | --- |
| Year | **Urban** | **Rural** |
| 1999 | 0.47 (0.44 - 0.51) | 0.29 (0.23 - 0.36) |
| 2000 | 7.53 (7.39 - 7.67) | 6.31 (6.04 - 6.58) |
| 2001 | 7.48 (7.34 - 7.62) | 5.85 (5.59 - 6.11) |
| 2002 | 7.41 (7.27 - 7.54) | 5.97 (5.71 - 6.24) |
| 2003 | 7.57 (7.43 - 7.71) | 6.18 (5.91 - 6.44) |
| 2004 | 7.68 (7.54 - 7.82) | 6.63 (6.36 - 6.9) |
| 2005 | 7.88 (7.75 - 8.02) | 6.79 (6.52 - 7.07) |
| 2006 | 7.64 (7.51 - 7.77) | 6.57 (6.3 - 6.83) |
| 2007 | 7.59 (7.45 - 7.72) | 6.52 (6.25 - 6.79) |
| 2008 | 7.39 (7.26 - 7.52) | 6.49 (6.23 - 6.75) |
| 2009 | 7.21 (7.08 - 7.33) | 6.18 (5.92 - 6.44) |
| 2010 | 7.18 (7.06 - 7.31) | 6.46 (6.2 - 6.72) |
| 2011 | 7.13 (7.01 - 7.26) | 6.5 (6.24 - 6.77) |
| 2012 | 6.83 (6.71 - 6.95) | 6.08 (5.83 - 6.33) |
| 2013 | 6.72 (6.6 - 6.84) | 6.27 (6.02 - 6.53) |
| 2014 | 6.59 (6.47 - 6.71) | 6.46 (6.2 - 6.72) |
| 2015 | 6.68 (6.56 - 6.8) | 6.19 (5.94 - 6.44) |
| 2016 | 6.6 (6.49 - 6.72) | 6.3 (6.04 - 6.55) |
| 2017 | 6.48 (6.37 - 6.59) | 6.42 (6.16 - 6.67) |
| 2018 | 6.48 (6.37 - 6.59) | 6.69 (6.43 - 6.95) |
| 2019 | 6.48 (6.37 - 6.59) | 6.41 (6.16 - 6.66) |
| 2020 | 6.8 (6.69 - 6.91) | 6.78 (6.52 - 7.04) |
